# Microplastic-induced multi-organ toxicity: cellular mechanisms and critical roles of organ crosstalk

**DOI:** 10.3389/fpubh.2026.1746924

**Published:** 2026-02-03

**Authors:** Lifang Zheng, Xiaojie Ma, Zhihai Jin, Zhijian Rao

**Affiliations:** 1 College of Physical Education, Shanghai University, Shanghai, China; 2 College of Physical Education, Shanghai Normal University, Shanghai, China; 3 Exercise Biological Center, China Institute of Sport Science, Beijing, China

**Keywords:** inflammatory response, microplastics, multi-organ toxicity, organ crosstalk, oxidative stress

## Abstract

Microplastics (MPs) are pervasive environmental contaminants with significant bioaccumulation potential, posing a growing threat to global health through multi-organ toxicity. This review systematically synthesizes current knowledge on MPs-induced organ-specific damage and its systemic health implications. We detail the accumulation of MPs in major organ systems, including the liver, brain, lungs, kidneys, intestines, heart, and reproductive organs. Furthermore, we emphasize the critical role of inter-organ communication in amplifying toxicity, such as gut-liver axis-mediated hepatotoxicity and gut-brain axis-driven neurotoxicity. Emerging evidence on the transgenerational adverse effects of parental MPs exposure is also discussed. The core cellular and molecular mechanisms across these organs are examined, with a particular focus on oxidative stress, inflammatory activation, mitochondrial dysfunction, and programmed cell death. This review is distinct in its integrative approach, offering a novel perspective by synthesizing organ-specific pathologies with cross-organ communication networks and transgenerational effects, thereby providing a more holistic understanding of MPs’ systemic toxicity. Collectively, this review elucidates the exposure-organ damage correlation, analyzes the underlying pathogenic mechanisms, and aims to provide a scientific foundation for public health risk assessment and informed environmental policy formulation.

## Graphical abstract

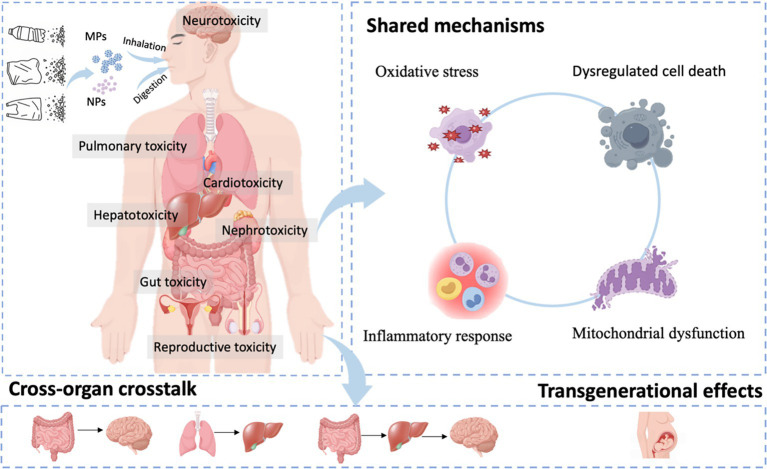

## 1 Introduction

With the continuous growth of global plastic production and consumption, microplastics (MPs, typically defined as synthetic polymer particles with diameters < 5 mm), an emerging and ubiquitous environmental pollutant, have been widely distributed in the hydrosphere, atmosphere, and soil. Human exposure occurs primarily through dietary intake, respiratory inhalation, and dermal absorption. Upon entering the body, MPs, along with the even more penetrative nanoplastics (NPs, typically defined as plastic particles with a size <1 μm), can distribute and accumulate in critical organs including the liver, intestines, kidneys, heart, lungs (1), where they elicit direct pathological responses such as oxidative stress, inflammatory cascades, metabolic dysregulation, and cellular dysfunction (2). Notably, NPs’ unique ability to traverse specialized biological barriers enables them to reach sensitive sites like the brain and fetus, posing direct risks for neurotoxicity (3), reproductive developmental abnormalities, and intergenerational toxicity (4).

The systemic health impact of MPs/NPs, however, extends beyond the sum of these localized effects. A growing body of research highlights their capacity to disrupt vital inter-organ communication axes, thereby amplifying and disseminating toxicity systemically. For instance, by compromising intestinal barrier integrity and altering gut microbiota, MPs can initiate inflammatory and metabolic signals that travel via the portal circulation to disrupt liver homeostasis, constituting the gut-liver axis (5). Similarly, disturbance of the gut-brain axis—through neural, endocrine, and immune pathways—is implicated in MPs-induced neurobehavioral abnormalities (6). Furthermore, the ability of NPs to cross the placental barrier directly establishes a placenta-fetus axis of exposure and potential developmental toxicity (7). This paradigm of “multi-organ crosstalk” is essential for understanding their systemic pathogenicity. Furthermore, both MPs and NPs can act as vectors for co-existing environmental contaminants (e.g., heavy metals, POPs), potentially amplifying adverse health effects through synergistic interactions (8, 9).

Critically, the translation of these mechanisms into tangible human health risks is an area of intense and rapidly evolving investigation. While evidence from *in vitro* and animal models is robust, large-scale human epidemiological data have been more limited until recently. A series of emerging cohort studies, however, are beginning to bridge this gap by providing critical human evidence (10, 11). Notably, a landmark 2023 cohort study published in *The New England Journal of Medicine (NEJM)* detected microplastics in human carotid artery plaques and reported a significant association with increased cardiovascular mortality risk (12). This study along with other contemporary epidemiological investigations (13), has been pivotal in shifting the perception of MPs/NPs from potential risk factors to clinically relevant toxicants, underscoring the urgent need for a comprehensive synthesis of existing evidence. Against this backdrop, this review systematically evaluates and synthesizes current biomedical evidence on MPs and NPs, encompassing their exposure pathways, biodistribution, and mechanistic toxicity spanning from organ-specific injury to inter-organ crosstalk. Ultimately, this synthesis aims to establish a scientific foundation for understanding the associated health risks and to inform evidence-based environmental and public health protection strategies.

Unlike previous reviews that primarily focus on environmental occurrence or single-organ toxicity of microplastics, this review provides an integrative framework linking exposure routes, multiorgan toxicity, cellular mechanisms, and inter-organ crosstalk. In particular, we emphasize emerging concepts such as the gut–liver–brain axis and transgenerational toxicity, offering a systems-level perspective on microplastics-induced health risks. The overall framework of microplastics-induced multiorgan toxicity, shared cellular mechanisms, and inter-organ crosstalk is schematically summarized in the graphical abstract.

## 2 Microplastics

### 2.1 Definition and source of microplastics

Plastics have become indispensable in modern industry and daily life due to their lightweight nature, corrosion resistance, and high mechanical stability. Since 1950, global plastic production has experienced exponential growth, reaching 348 million tons in 2017, with projections exceeding 3.3 billion tons by 2050 (14). However, effective end-of-life management of plastic products remains a critical global challenge. Despite service lifetimes spanning 1–50 years, current recycling systems capture merely 9% of plastic waste for energy recovery, with only 12% undergoing material recycling. Alarmingly, 8% contaminates terrestrial ecosystems while the majority (71%) is released uncontrolled into the environment, creating persistent pollution (15). It is estimated that the total amount of plastic released into the environment globally each year exceeds 1.5 million tons, with tire wear (28%), urban dust (24%), and textile washing (15%) being the main contributing sources (16). Environmental plastic pollution is predominantly composed of synthetic polymers such as polypropylene (PP), polyethylene (PE), polyethylene terephthalate (PET), polystyrene (PS), polyurethane (PUR), polyvinyl chloride (PVC), and polycarbonate (PC) (17). These polymeric materials undergo progressive environmental degradation through synergistic effects of ultraviolet radiation, chemical oxidation, and mechanical weathering, ultimately fragmenting into size-fractionated plastic debris categorized as: macroplastics (>25 mm), mesoplastics (5–25 mm), microplastics (0.1 μm-5 mm), and nano-plastics (<0.1 μm) (17). Current scientific consensus on microplastic size classification remains debated: Frias and Nash originally defined them as insoluble synthetic solid particles measuring 1–5 mm (18), while more inclusive classification schemes extend the upper size limit to 5 mm (19). In accordance with prevailing conventions, this review operationally defines MPs as plastic particulates with diameters < 5 mm, encompassing the full spectrum of sub-5 mm synthetic polymer fragments. Based on their origin, MPs are classified into two distinct categories: primary MPs and secondary MPs. Primary MPs are intentionally manufactured industrial precursors, including cosmetic microbeads, textile fibers, and raw resin pellets. Secondary MPs result from the progressive environmental degradation of larger plastic items such as packaging materials, fishing nets, and vehicle tires through weathering processes (18). Notably, secondary MPs form through synergistic environmental interactions (photochemical aging, biofilm colonization, hydrodynamic abrasion), which modify their surface properties, including an increased specific surface area and oxidative functionalization. These changes increase the adsorption capacity of toxic contaminants (heavy metals, persistent organic pollutants), ultimately posing substantial risks to ecosystem health through food chain amplification.

### 2.2 Physical and chemical properties of microplastics

MPs are pollutants widely present in the environment, and their physicochemical properties directly affect their interactions with living organisms. The size, shape, and surface characteristics of MPs determine their biocompatibility and the complexity of their biological interactions. The size of MPs is a key factor in their biological penetration ability. Emerging evidence indicates that nano-plastics (<1 μm) exhibit enhanced cellular membrane penetration and biological barrier translocation due to their ultrafine dimensions, consequently inducing cellular damage and systemic responses (20). Furthermore, the shape of MPs greatly affects their biological interactions. Irregularly shaped MPs demonstrate greater mechanical cytotoxicity than spherical counterparts, likely attributable to edge-induced physical disruption during cellular contact (21). The surface characteristics of MPs, particularly their charge distribution and functional group composition, are critical determinants of their interactions with biological systems. Specifically, negatively charged MPs exhibit preferential binding affinity for cationic biomolecules through electrostatic interactions, which can subsequently affect cellular function and biological reactions (22). In addition, MPs can also serve as carriers of pollutants, and this increases their environmental and biological hazards. Research has found that MPs can effectively absorb persistent organic pollutants (POPs) and heavy metals, forming complexes that may lead to more severe toxic effects upon entering living organisms (20). Additives in MPs, such as plasticizers, may leach into the environment, resulting in synergistic toxicity effects that exacerbate harm to organisms. The surface of MPs may also form biofilms, which can promote the spread of pathogens and make MPs a potential vector for disease transmission, posing a threat to ecosystems and human health.

### 2.3 Distribution and organ accumulation of microplastics

Initially, research predominantly focused on the ecological impacts of MPs. However, recent studies have detected MPs within human tissues and organs, suggesting potential health hazards to humans. Current research identifies dietary intake, water consumption, inhalation, and dermal contact as the primary pathways for human microplastic uptake (23). Specifically, orally ingested MPs traverse the digestive system, eventually reaching the intestines where they may be absorbed by intestinal epithelial cells. This absorption process is not only dependent on the microplastics’ particle size and chemical properties but is also highly contingent upon the host’s physiological state. Studies have demonstrated that fluorescently labeled polystyrene nanoparticles accumulated and aggregated within the digestive systems of mice, disseminating to multiple organs and profoundly impairing cellular function (2). Furthermore, MPs could potentially enter the systemic circulation via intestinal permeability, reaching major target organs such as the liver and inducing systemic biological effect (5). Additionally, MPs can be inhaled through the respiratory system. Ultrafine plastic particles present in the air may enter the lungs during breathing and deposit within lung tissue. Research has shown that such pulmonary deposition can not only cause mechanical obstruction but also trigger localized inflammatory responses and oxidative stress, thereby compromising pulmonary function and overall health (24). Recent studies reveal that nanoscale plastics (<0.1 μm) can penetrate biological barriers – such as the blood–brain barrier and placental barrier – inducing oxidative stress and mitochondrial dysfunction. For instance, experimental evidence demonstrates that polystyrene nanoparticles administered to mice traverse these barriers, accumulating in the brain and placenta where they trigger apoptosis and inflammatory responses (25). Current research has identified that MPs accumulate primarily in vital organs including the circulatory system, liver, kidneys, lungs, heart, and brain (1). Hepatic accumulation of MPs significantly contributes to impaired metabolic functions, while MPs induced renal damage can compromise electrolyte balance and detoxification capabilities. Furthermore, pulmonary deposition of MPs has been demonstrated to induce chronic inflammation and oxidative stress, thereby compromising respiratory health. Within the brain, accumulating MPs show potential links to the pathogenesis of neurodegenerative disorders. These organ-specific accumulation profiles underscore the necessity of investigating particulate distribution patterns and mechanisms of action when evaluating the health impacts of MPs.

## 3 Organ damage induced by microplastics

Once regarded as biologically inert particles, MPs are now recognized as posing significant hazards to living organisms. Following their entry into the human body, MPs disseminate into systemic circulation and peripheral tissues. Their high surface area-to-volume ratio and environmental persistence may induce oxidative stress, chronic inflammation, and cytotoxicity, ultimately impairing tissue function. Substantial evidence demonstrates that MPs elicit multi-organ toxicity affecting the liver, intestines, kidneys, heart, brain, and reproductive system, compromising physiological functions across these organ systems. This section synthesizes current preclinical evidence to systematically examine the potential adverse effects of microplastics on organ pathophysiology.

### 3.1 Hepatotoxicity

MPs accumulate in and induce damage within the livers of fish, mammals, and cirrhotic patients (26, 27). The exposure of mice to 0.1 μm MPs (1 mg/L, 60 days) induces hepatocytic mtDNA lesions and hepatic fibrosis (28). Zou et al. (29) demonstrated that exposure to 5 μm and 0.5 μm MPs (10 mg/L, 3mouths) reduces the liver-to-body weight ratio in mice, concurrently disrupting hepatic architecture and inducing nuclear pyknosis and mitochondrial vacuolization. Similarly, exposure to 5 μm MPs (0.5 mg/100 μL) for four weeks significantly elevated serum alanine aminotransferase (ALT) and aspartate aminotransferase (AST) levels in mice, while enhancing intrahepatic natural killer (NK) cell activity and macrophage infiltration (30). These collective findings demonstrate microplastic-induced hepatic injury.

Hepatic fibrosis represents the pathological endpoint of recurrent liver injury and repair processes. Studies demonstrate that small particle size MPs (0.1 μm) activate the hepatic cyclic GMP-AMP synthase/stimulator of interferon genes (cGAS/STING) signaling pathway in mice, driving nuclear factor kappa B (NF-κB) nuclear translocation, upregulating pro-inflammatory cytokines [interleukin-6 (IL-6), interleukin-1β (IL-1β), tumor necrosis factor-α (TNF-α)], and ultimately promoting fibrogenesis (28). Additionally, MPs induce macrophage extracellular trap macrophage extracellular trap (MET) formation, subsequently activating the reactive oxygen species (ROS)/transforming growth factor-β (TGF-β)/Mothers against decapentaplegic homolog 2/3 (Smad2/3) axis to drive inflammatory responses and hepatocyte epithelial-mesenchymal transition (EMT), thereby accelerating fibrosis progression (31). Three-dimensional liver organoid (LO) models reveal that microplastic exposure (1 μm, 25 μg/mL, 2.5 μg/mL and 0.25 μg/mL,48 h) downregulates fatty acid oxidation genes (carnitine palmitoyltransferase-1α, CPT-1α) while upregulating lipogenesis genes (acetyl-CoA carboxylase 1, ACC1), causing abnormal lipid accumulation and suggesting potential induction of hepatic steatosis and fibrosis (32). Furthermore, MPs instigate oxidative stress by suppressing sirtuin 3 (SIRT3) and superoxide dismutase 2 (SOD2) expression. This oxidative cascade disrupts mitochondrial membrane potential and suppresses respiratory chain activity, culminating in hepatocyte damage (33). These findings establish oxidative stress and mitochondrial dysfunction as key mechanisms of microplastic-induced hepatocyte injury. Critically, MPs exposure triggers hepatic Ca^2+^ overload, activating AMP-activated protein kinase-peroxisome proliferator-activated receptor gamma coactivator 1-alpha (AMPK-PGC-1α) signaling and enhancing glycolytic flux to induce hepatocyte apoptosis (34). Particle size dictates distinct cell death pathways: smaller MPs (1-10 μm, 10 mg/L, 30 days) induce necroptosis via the phosphatase and tensin homolog/phosphoinositide 3-kinase/protein kinase B (PTEN/PI3K/AKT)/autophagy axis, while larger MPs (50-100 μm, 10 mg/L, 30 days) promote apoptosis through the same pathway, with autophagy inhibition partially rescuing these phenotypes (35). Collectively, MPs drive hepatotoxicity through concerted pathways involving oxidative damage, metabolic disruption, and dysregulated cell death programs (Table 1).

**Table 1:** Comprehensive summary of microplastic induced hepatotoxicity.

Type of MP	Exposure route	Size/Dose/Times	Detected effect	Study model and analytical techniques
PS-MPs (28)	OD	0.1 μm, 1 mg/L, 60 days	Liver injury (↑ ALT and AST significantly); Oxidative stress markers: (↓ MDA and GSH, graph only, no exact fold reported); Liver fibrosis (↑ α-SMA and fibronectin, not quantified); Mitochondrial dysfunction (↓ ATP, mtDNA leakage, graphical data); Inflammation (cGAS/STING pathway activated, cytokine expression ↑: (IL-1β, IL-6, TNF-α), graph only, no exact fold reported).	*In vivo*; Fluorescence microscopy
PS-MPs (29)	OD	5 μm, 10 mg/L, 3 months	Liver injury, not quantified; Oxidative stress markers: ↑ MDA and ↓ T-AOC, SOD, CAT, GSH, graph only, no exact fold reported.	*In vivo*; Not applicable
PS-MPs (30)	OD	5 μm, 0.5 mg/100 μL, 4 weeks	Liver injury (↑ ALT, AST, TBIL); NK cells activated and macrophages infiltration significantly ↑ (↑ F4/80 and CD11, ↓ CD206), graph only, no exact fold reported; NF-kB pathway activated, Inflammation cytokine expression (↑IFN-γ, TNF-α, IL-1β, IL-6, IL-33 and ↓ IL-4, IL-5, IL-10, IL-18, TGF-β1), graph only, no exact fold reported.	*In vivo*; Not applicable
PS-MPs (31)	OD	1-10 μm, 10 mg/L, 30 days	Liver fibrosis, graphical data; EMT (↑ α-SMA, FSP1, FN1, Vimentin and ↓ E-cadherin) and Inflammation cytokine expression (↑ TNF-α, IL-1, IL-6, IL-8 and ↓ IL-10), graph only, no exact fold reported; Macrophage infiltration significantly ↑ by 10.04%, METosis formation, graphical data; TGF-β/Smad2/3 pathway activated, graph only, no exact fold reported.	*In vivo* and *vitro*; Not applicable
PS-MPs (34)	OD	4.98 ± 0.32 μm, 1 mg/L, 30 days	Ca^2+^ overload and ROS level significantly ↑, graphical data;	*In vivo*; Not applicable
			AMPK/PGC-1α pathway activated, graph only, no exact fold reported.	
PS-MPs (35)	OD	1-10 μm and 50-100 μm, 10 mg/L, 30 days; 500 μg/L f-or 24 h	Apoptosis (↓ Bcl-2 and ↑Bax, CAS3, cle-CAS3) and necroptosis (↑ RIP1, RIP3, MLKL) significantly ↑, graph only, no exact fold reported; PTEN/PI3K/Akt pathway inhibited (↑PTEN, ↓PI3K, Akt); Autophagy activated (↑ Becn1, ATG7, LC3 and ↓ P62), graph only, no exact fold reported.	*In vivo* and *vitro*; Not applicable

Micro-PS, microplastics; PS-MPs, polystyrene microplastics; OD, oral (drinking); cGAS, cyclic GMP-AMP synthase; STING, stimulator of interferon genes; EMT, epithelial-mesenchymal transition; ROS, reactive oxygen species; TGF-β, transforming growth factor-β; AMPK, adenosine 5′-monophosphate (AMP)-activated protein kinase; PGC-1α, peroxisome proliferator-activated receptor g coactivator 1-alpha; PTEN, phosphatase and tension homolog; PI3K, phosphatidylinositol 3-kinase; Akt, protein kinase B; ALT, Alanine Aminotransferase; AST, Aspartate Aminotransferase; NF-kB, Nuclear factor kappa-B; α-SMA, α-smooth muscle actin; IL-1β, interleukin-1β; IL-6, interleukin-6; SOD, superoxide Dismutase; MDA, malondialdehyde; CAT, catalase; Bax, BCL-2-associated X protein; Bcl-2, B-cell lymphoma-2; LC3, microtubule-associated protein 1 light chain 3; ATG7, autophagy-related gene 7; MLKL, mixed lineage kinase domain-like; RIP3, receptor-interacting protein kinase 3; RIP1, receptor-Interacting protein kinase 1; cle-CAS3, cleaved caspase-3; CAS3, caspase-3; IL-8, interleukin-8; FN1, fibronectin 1; FSP1, ferroptosis suppressor protein 1; IL-18, interleukin-18; IL-10, interleukin-10; IL-5, interleukin-5; IL-4, interleukin-4; IL-33, interleukin-33; INF-γ, interferon-gamma; T-AOC, total antioxidant capacity; ↑Increase or enhance; ↓Reduce or inhibit.

### 3.2 Neurotoxicity

MPs particles translocating through the gastrointestinal and respiratory tracts can cross the blood–brain barrier into the central nervous system, eliciting neurotoxic effects (36, 37). MPs (5 μm, 0.01, 0.1 and 1 mg/day for 4 weeks) exposure compromises learning and memory in mice, characterized by disorganized hippocampal neurons, reduced Nissl bodies, elevated ROS and malondialdehyde (MDA) levels, decreased glutathione and acetylcholine, and significant suppression of the cAMP response element-binding protein/brain-derived neurotrophic factor (CREB/BDNF) signaling pathway (38). Furthermore, MPs (100 nm) exposure induces neuronal apoptosis while downregulating synapse-associated protein markers (39). Exposure to MPs triggers reactive astrogliosis and significantly elevates the expression of lipocalin-2 (LCN2), a secreted neurotoxic protein, in activated astrocytes, thereby driving paracrine neuronal degeneration (40). Thus, MP-induced neurotoxicity may arise either from direct cellular stress in MP-internalizing neurons, or from the release of neurotoxin (e.g., LCN2) by adjacent reactive astrocytes. Furthermore, MPs exhibit strong iron-binding capacity. Co-exposure to MPs and iron induces cerebral iron overload and cognitive deficits in aged mice, significantly promoting ferroptosis—an iron-dependent cell death pathway driven by lipid peroxidation and neuroinflammation (9). This indicates that iron-microplastic synergism may exacerbate cognitive impairment by disrupting cerebral iron homeostasis and inducing ferroptosis in cognition-related brain regions. This synergy highlights a specific and potent mechanism through which MPs can amplify the toxicity of environmental pollutants.

Beyond acute neurotoxicity, MPs exposure may also exacerbate molecular hallmarks associated with neurodegenerative diseases. Reduced acetylcholinesterase (AChE) activity is a feature observed in pathologies neurodegenerative such as Alzheimer’s and Parkinson’s diseases (41). Similarly, MPs exposure has been shown to suppresses AChE activity across multiple species (3), suggesting a potential for MP to modulate cholinergic neurotransmission in a manner reminiscent of neurodegenerative pathogenesis. Consistent with this, Liang et al. demonstrated that MPs induce Parkinson’s disease-like neurodegeneration in mice, characterized by disrupted energy metabolism in the substantia nigra pars compacta (SNC) and striatum—evidenced by diminished adenosine triphosphate (ATP) levels and downregulation of ATP-associated genes/proteins (42), This indicates MPs may drive neurodegeneration via impaired.

The neurotoxic potential of MPs is further modulated by their physical properties and exposure routes, highlighting a critical dimension of risk assessment. Studies reveal enhanced cellular uptake of smaller MPs (80 nm) compared to their larger counterparts (100 or 200 nm) (37). Moreover, the exposure route significantly impacts outcomes; for instance, aerosol-inhaled MPs (80 nm for 7 days) cause greater locomotor deficits in mice than exposure via drinking water (37). These findings suggest inhaled MPs, particularly in the nanoscale range, may pose a heightened neurotoxic risks due to more efficient pulmonary translocation and systemic distribution.

Collectively, these findings demonstrate that MPs induce neurotoxicity through multifaceted mechanisms involving oxidative stress, suppression of neurotrophic signaling (e.g., CREB/BDNF), induction of specific cell death pathways (e.g., ferroptosis), and potential exacerbation of neurodegenerative phenotypes. They also act as vectors for other neurotoxicants like iron. Furthermore, their toxicity is influenced by particle size and exposure route. Despite the established neurotoxicity in experimental models, critical gaps persist regarding the precise mechanisms of MPs translocation to the brain, their capacity to breach the blood–brain barrier under chronic, low-dose scenarios, and their role in initiating or accelerating human neurodegenerative disorders (Table 2).

**Table 2:** Comprehensive summary of microplastic induced neurotoxicity.

Type of MP	Exposure route	Size/Dose/Times	Detected effect	Study model and analytical techniques
PS-MPs (38)	OG	5 μm, 1 mg/day, 4 weeks	Learning and memory significantly ↓; ROS level significantly ↑ (graph only, no exact fold reported); CREB/BDNF pathway inhibited (graph only, no exact fold reported).	*In vivo*; Not applicable
PS-MPs (9)	OD	5 μm, 1,000 μg/L, 3momths	Cognitive capacity significantly ↓; Iron content and ferroptosis significantly ↑ (GPX4 ↓), graph only, no exact fold reported; Inflammatory significantly ↑ (↑ GFAP, Iba1 and IL-1β, graph only, no exact fold reported).	*In vivo*; Inductively coupled plasma mass spectrometry
PS-NPs (42)	OG	50 nm, 0.25–250 mg/kg, 28 days	PD-like neurodegeneration ↑(Blood brain barrier integrity compromised), graph only, no exact fold reported; Neurons mitochondrial dysfunction and energy metabolism disorder (graphical data).	*In vivo*; Panoramic MIDI

Micro-PS, microplastics; PS-MPs, polystyrene microplastics; PS-NPs, polystyrene nanoplastics; OD, oral (drinking); OG, oral gavage; ROS, reactive oxygen species; CREB, cyclic adenosine monophosphate response element binding protein; BDNF, brain derived neurotrophic factor; PD, Parkinson’s disease; GPX4, glutathione peroxidase 4; GFAP, glial fibrillary acidic protein; Iba1, ionized calcium-binding adapter molecule 1; ↑, increase or enhance; ↓, reduce or inhibit.

### 3.3 Pulmonary toxicity

MPs accumulate in the respiratory system via inhalation, exhibiting significant size-dependent pulmonary toxicity. Clinical evidence confirms MPs in human upper and lower respiratory tracts—including alveoli, lung tissues, and sputum from patients with respiratory diseases—with inhaled MPs constituting the primary source of pulmonary deposition (43–45). Animal studies demonstrate that chronic exposure to both small (1–5 μm,20 μL, 3 weeks) and large (10-20 μm, 20 μL, 3 weeks) MPs induces severe lung injury, characterized by robust inflammatory responses, elevated cellular apoptosis, and pathological collagen deposition (46). Notably, smaller MPs provoke significantly more severe parenchymal damage (46). This indicates that the toxic effects of MPs are closely related to the exposed particle size. Small particle size MPs, due to their larger specific surface area and penetration ability, are more likely to trigger oxidative stress and inflammatory cascade reactions (46, 47).

MPs drive pulmonary injury through multi-pathway disruption of cellular homeostasis. Microplastics activate the NOD-like receptor protein 3 (NLRP3) /caspase-1/IL-1β and Toll-like receptor 3(TLR3) /NF-κB signaling axes, driving the release of pro-inflammatory factors (IL-6, TNF-α, IL-1β), leading to alveolar destruction, disordered arrangement of bronchial epithelium, and lung tissue damage (46, 48). *In vitro* evidence using murine alveolar epithelial cells (MLE-12) confirms MPs (100 nm, 5 mg/kg, three times/week for a week) exposure activates inflammasomes, elevating pyroptosis, ROS and pro-inflammatory mediators (TNF-α, IL-6, IL-1β), suggesting that MPs-induced lung injury by inflammasome activation, oxidative stress, and inflammation (48). Furthermore, MPs promote cellular senescence in human pulmonary epithelial cells and murine lungs via ROS-dependent pathways (24). Exposure to MPs induces pulmonary dysfunction in Sprague–Dawley (SD) rats, accompanied by infiltration of inflammatory cells and upregulation of aging related markers (p21, p16, p27), resulting in a decline in lung tissue repair function, suggesting that cellular aging may be a key phenotype of MPs induced lung injury (49). Emerging evidence suggests MPs may disrupt respiratory microbiome homeostasis and dysregulate non-coding RNAs (e.g., abnormal expression of 269 circular RNAs (circRNAs) and 109 long non-coding RNAs (lncRNAs)) during lung injury progression (50, 51). However, the epigenetic regulatory mechanisms underlying these effects require further elucidation. In summary, MPs compromise pulmonary integrity through interconnected pathways involving inflammatory activation, oxidative damage, and accelerated cellular senescence (Table 3).

**Table 3:** Comprehensive summary of microplastic induced pulmonary toxicity.

Type of MP	Exposure route	Size/Dose/Times	Detected effect	Study model and analytical techniques
PS-MPs (46)	IN	1-5 μm and 10-20 μm, 20 μL, 3 weeks；100 μg/mL, 250 μg/mL, 500 μg/mL for 48 h	Lung injury (↑ Collagen deposition); ↑ ROS fluorescence intensity; Inflammation cytokine expression: (↑ NLRP3, caspase-1, IL-1β, IL-6, TNF-α); Apoptosis markers: (↑ caspase-3, caspase-9 and Bax), graph only, no exact fold reported; Lung fibrosis (↓ E-cadherin, ↑ MMP-1, MMP-2, MMP-9); TLR2/NF-kB pathway activated, graph only, no exact fold reported.	*In vivo* and *vitro*; Not applicable
APS-NPs (48)	IT	100 nm, 5 mg/kg, 3x/k, 7 days	Lung injury (graphical data); ROS and Inflammation markers ↑ (↑MDA, IL-6, TNF-α and IL-1β), graph only, no exact fold reported; Proptosis (NLRP3/capase-1/IL-1βpathway activated, graph only, no exact fold reported)	*In vivo* and *vitro*; Not applicable
PS-MPs (49)	INH	100 nm, 0.5, 1.0 or 2.0 mg/m^3^, four hours/day, 35 days	Lung dysfunction and inflammatory injury (↑ IL-6 and IL-8) graph only, no exact fold reported; Cellular senescence makers: (↑ p21, p16 and p27 significantly, graph only, no exact fold reported).	*In vivo*; Fluorescence microscopy imaging
PS-MPs and PS-NPs (50)	IN	5 μm MP 99 nm NP, 10 μg/μl, 5 weeks,	Nasal and lung microbial dysbiosis (graphical data).	*In vivo*; Not applicable
PS-MPs (51)	IT	100 nm, 0.5, 1, or 2 mg/200 μL, 14 days	Lung injury ↑ (graphical data); Inflammation cytokines expression (↑ IL-6, TNF-α, IL-1β), graph only, no exact fold reported.	*In vivo*; Fluorescence microscopy imaging

PS-MPs, polystyrene microplastics; PS-NPs, polystyrene nanoplastics; IN, intranasal administration; IT, intratracheal instillation; INH, inhalation; PVC, polyvinyl chloride; ROS, reactive oxygen species; NLRP3, NOD-like receptor family pyrin domain containing 3; Caspase-1, cysteine-aspartate protease 1; IL-1β, interleukin-1β; IL-6, interleukin-6; TNF-α, tumour necrosis factor alpha; Caspase-3, cysteine-aspartate protease 3; Caspase-9, cysteine-aspartate protease 9; Bax, Bcl-2-associated X protein; MMP-1, matrix metalloproteinase 1; MMP-2, matrix metalloproteinase 2; MMP-9, matrix metalloproteinase 9; TLR2, Toll-like receptor 2; NF-kB, nuclear factor kappa-B; MDA, malondialdehyde; IL-8, interleukin-8; ↑Increase or enhance; ↓Reduce or inhibit.

### 3.4 Nephrotoxicity

As the central organ regulating hydroelectrolyte balance and metabolic waste excretion, the kidney represents a critical target for MPs toxicity. Studies confirm MPs accumulation in mammalian kidneys, where exposure (100 nm, 25 mg/kg/d for 6 weeks) disrupts serum biomarkers (urea nitrogen [BUN], creatinine [CRE]) and pro-inflammatory mediators, inducing renal injury in juvenile rats (52). MPs exposure compromises renal antioxidant defenses by suppressing superoxide dismutase (SOD), glutathione peroxidase (GSH-Px), and catalase (CAT) activities, while elevating MDA and ROS—thus disrupting redox homeostasis (52, 53). Oxidative stress activates the NF-κB/NLRP3 inflammasome axis, amplifying pro-inflammatory cytokines (interleukin-18 (IL-18), IL-1β) that drive renal inflammatory infiltration and tissue damage (54).

Moreover, MPs dysregulate autophagy via the ROS/AMPK/Unc-51-like autophagy activating kinase 1 (ULK1) pathway (55), while synergizing with endoplasmic reticulum (ER) stress to exacerbate mitochondrial dysfunction (e.g., elevated Bad protein, membrane potential depolarization) and autophagosome accumulation to induce renal damage (56, 57). Concurrently, MPs exposure promotes renal apoptosis, as evidenced by an increased proportion of terminal deoxynucleotidyl transferase dUTP nick end labeling (TUNEL)-positive cells and upregulation of apoptosis-related genes (B-cell lymphoma 2 (Bcl-2), Bcl-2-associated X protein (Bax), Caspase-3, Caspase-9, Caspase-12) (58). Chronic MPs exposure promotes ferritinophagy-dependent iron accumulation, suppresses glutathione metabolism, and elevates lipid peroxidation markers (4-hydroxynonenal (4-HNE), 8-hydroxy-2′-deoxyguanosine (8-OHdG)), which synergize with transforming growth factor-β (TGF-β) secretion to induce renal fibrosis (57, 59). Critically, MPs act as contaminant vectors, co-amplifying cadmium-induced oxidative damage through Parkin/autophagy-related 5 (Parkin/ATG5)-dependent mitophagy (57). In short, MPs-induced nephrotoxicity involves interconnected networks of oxidative stress, inflammation, cell death, and autophagic dysregulation. Future research must establish chronic exposure thresholds and elucidate co-contaminant synergism mechanisms (Table 4).

**Table 4:** Comprehensive summary of microplastic induced nephrotoxicity.

Type of MP	Exposure route	Size/Dose/Times	Detected effect	Study model and analytical techniques
PS-NPs (52, 58)	OG	100 nm, 1.05 g/cm^3^, 25 mg/kg/day, 6 weeks	Lipid deposition ↑ (TG, TC and NEFA levels), graph only, no exact fold reported; Inflammation significantly ↑ (↑ IL-6: 24.39 ± 0.05%, IL-1β: 20.99 ± 1.46%, TNF-α: 41.55 ± 2.41%, ↓ IL-10: 35.62 ± 6.35%); Oxidative stress markers: (↓ SOD: 24.60 ± 1.28%, CAT: 26.35 ± 3.12%, GSH-Px: 35.77 ± 0.45%, ↑ MDA: 24.30 ± 4.95%).	*In vivo*; Not applicable
PS-MPs(53)	OG	2 μm, 0.2 or 0.4 mg/dose, 2x/week × 4 or 8 weeks; 0.05, 0.1, 0.2, 0.4, or 0.8 mg/mL for 1 or 2 h	Kidney injury (↓ serum creatinine levels), ER stress↑ (IRE1α), Inflammation↑ (COX1), Autophagy ↑ (LC3), not quantified.	*In vitro* and *vivo*; Raman spectroscopy
PS-MPs (55)	OD	1-10 μm, 10 mg/L; 300 μg/mL for 24 h	Oxidative stress markers: (↑ MDA, ↓ CAT, T-AOC, GSH-Px, SOD and GSH, graph only, no exact fold reported); Mitochondrial dysfunction (↑PGC-1α, ↑Mfn2, ↓Drp1, graph only, no exact fold reported); Autophagy significantly ↑ (LC3B, Beclin1, p60, Atg5 and Atg2, graph only, no exact fold reported).	*In vitro* and *vivo*; Not applicable
PS-MPs (58)	OG	1 μm, 2.0 mg/kg/, 28 days	Oxidative stress markers: (MDA significantly increased by 0.14 nmol/mg, SOD and GSH-Px significantly ↓); Inflammation significantly ↑: TNF-α (28.8%), IL-6 (19.70%) and IL-1β (37.32%), graph only, no exact fold reported; Apoptosis (↑ Bax, Caspase-3, Caspase-9, Caspase-12 and ↓ Bcl-2), graph only, no exact fold reported; ER markers ↑ (GRP78、IRE1、XBP1s、ATF6、JNK and CHOP).	*In vivo*; Not applicable
PS-MPs, PS-NH2 MPs (59)	OD	1.5 mg/kg/day, 6 months	Pro-inflammatory (↑ CXCL1, IL-1β, IL-6 and TNF-α) and anti-inflammatory (↓ IL-4, IL-10 and IL-13) factors expression, graph only, no exact fold reported; ROS significantly ↑ (MDA ↑ and SOD, GSH-Px, GSH/GSSG ↓) graph only, no exact fold reported; Ferroptosis markers ↑ (ACSL4, PTGS2 and NOX1) graph only, no exact fold reported.	*In vivo*; Quantitative fluorescence and bioluminescence imaging system
PS-NPs (60)	OG	300 nm, 1 mg/kg, 35 days	Antioxidant markers (SOD, GSH-Px and Nrf2 ↓, graph only, no exact fold reported); Iron mortality (↓ SLC7A11, GPX4 and FTH1; ↑ PTGS2, FTL and HMGB1, graph only, no exact fold reported); Excessive Mitophagy (↓ p62; ↑Parkin, Pink1, ATG5, LC3 and LC3B, graph only, no exact fold reported).	*In vivo*; Not applicable

PS-MPs, polystyrene microplastics; PS-NPs, polystyrene nanoplastics; OD, oral (drinking); OG, oral gavage; ROS, reactive oxygen species; ER, endoplasmic reticulum stress; Nrf2, nuclear factor erythroid 2-related factor 2; IL-1β, interleukin-1β; IL-6, interleukin-6; Bax, BCL-2-associated X protein; Bcl-2, B-cell lymphoma-2; TNF-α, tumor necrosis factor alpha; TG, triglycerides; TC, total cholesterol; NEFA, non-esterified fatty acids; IL-10, interleukin-10; SOD, superoxide dismutase; CAT, catalase; GSH-Px, glutathione peroxidase; MDA, malondialdehyde; IRE1α, inositol-requiring enzyme 1 alpha; COX-1, cyclooxygenase 1; LC3, microtubule-associated protein 1 light chain 3; T-AOC, total antioxidant capacity; GSH, glutathione; Ppargc1α, peroxisome proliferator-activated receptor gamma coactivator 1 alpha; Mfn2, mitofusin 2; Drp1, dynamin-related protein 1; Atg5, autophagy-related gene 5; Atg2, autophagy-related gene 2; GRP78, glucose-regulated protein 78; XBP1s, x-box binding protein 1 spliced; ATF6, activating transcription factor 6; JNK, c-Jun N-terminal kinase; CHOP, C/EBP homologous protein; CXCL1, Chemokine (C-X-C motif) ligand 1; IL-4, interleukin-4; IL-13, interleukin-13; GSSG, oxidized glutathione; ACSL4, acyl-CoA synthetase long-chain family member 4; PTGS2, prostaglandin-endoperoxide synthase 2; NOX1, NADPH oxidase 1; SLC7A11, solute carrier family 7 member 11; GPX4, glutathione peroxidase 4; FTH1, ferritin heavy chain 1; FTL, ferritin light chain; HMGB1, high mobility group box 1; LC3B, microtubule-associated protein 1 light chain 3 beta; Pink1, phosphatase and tensin homolog-induced putative kinase 1; ↑increase or enhance; ↓reduce or inhibit.

### 3.5 Gut toxicity

The intestinal toxicity of MPs is closely related to their size dependent absorption: particles >150 μm adhere to the intestinal mucus layer or directly interact with epithelial cells, triggering intestinal inflammation, whereas particles <150 μm penetrate the mucus barrier for subsequent intestinal absorption (60). Clinical evidence reveals significantly elevated fecal MPs loads in inflammatory bowel disease (IBD) patients compared to healthy individuals (up to 1.5-fold higher), with concentrations positively correlated with symptom severity including diarrhea and rectal bleeding (61). MPs enrichment was observed in both colon resection samples (62) and colitis model mice, accompanied by shortened colon length, increased permeability and inflammation, and reduced mucus secretion, suggesting that it may promote disease progression by exacerbating intestinal barrier damage (62, 63).

MPs induce intestinal toxicity through multiple pathways. Studies demonstrate that MPs exposure suppresses the expression of mucus secretion-related genes (Mucin 1(Muc1), Krüppel-like factor 4 (Klf4)) and ion transporters (cystic fibrosis transmembrane conductance regulator (CFTR), Na^+^-K^+^-2Cl^−^ cotransporter 1 (NKCC1), Na^+^/H^+^ exchanger 3 (NHE3)), thereby compromising the mucus barrier and inducing electrolyte imbalances (64, 65). In parallel, MPs exposure elevates (ROS) production. Excessive ROS further mediates intestinal epithelial cell apoptosis (66) and, by inhibiting methyltransferase-like 3 (METTL3) expression, reduces the mRNA stability of pro-angiogenic factors, impeding intestinal angiogenesis (67). Furthermore, as the gut microbiota serves as a pivotal mediator of metabolic and immune regulation, MPs disrupt intestinal homeostasis by altering microbial composition (e.g., causing an imbalance in the Firmicutes/Bacteroidetes ratio) and modulating microbial metabolite secretion (68, 69). Notably, exposure to high-dose (200 μg/g), large-size MPs (10–150 μm) for 5 weeks upregulates Toll-like receptor 4 (TLR4), activator protein 1(AP-1), and interferon regulatory factor 5 (IRF5) expression, driving immune cell infiltration in the colon and duodenum (70). Conversely, short-term (4 weeks) exposure to low-dose (≤ 34 mg/kg), small-size MPs (1–10 μm) did not elicit significant inflammatory responses in the duodenum or colon (71). This suggests that the toxicological effects of MPs are dynamically regulated by particle size, dose, and exposure duration. Moreover, pre-existing pathological conditions (e.g., susceptibility to colitis) may amplify injury risks. These contrasting findings highlight the imperative need to establish standardized exposure models and integrate multi-omics analyses (Table 5).

**Table 5:** Comprehensive summary of microplastic induced gut toxicity.

Type of MP	Exposure route	Size/Dose/Times	Detected effect	Study model and analytical techniques
PS-MPs (64)	OD	0.5 or 50 μm, 100 or 1,000 μg/L, 5 weeks	Gut microbiota dysbiosis, and hepatic lipid disorder (↓PK, PPARα, FAS and ↑Chrebp, Fat1, Fatp2, CS, PPAR*γ*, ACC, ACL, Dgat1, Dgat2, Gpat), graph only, no exact fold reported.	*In vivo*; Not applicable
PS-MPs (65)	OD	5 μm, 100 and 1,000 μg/L, 6 weeks	Gut microbiota dysbiosis (↓ Parabacteroides, Prevotella, Dehalobacterium, Turicibacter, Bifidobacterium, Lachnospira, Haemophilus, Adlercreutzia, Megamonas, Blautia, Dialister, and↑ Coprococcus, Anaeroplasma); Intestinal barrier dysfunction (↓Muc1、Muc2、Klf4 and Retnlb); Metabolic disorders (↑ ARG, TYR, C4, C5, C6DC, C16, C18, and ↓ LEU/ILE/PRO-OH, ORN, PHE, PRO, VAL).	*In vivo*; Confocal microscope
PS particles (66)	OG	50 nm, 0.5 μm, 5 μm, 1.05 g/mL, 20 mL/kg, 28 days	Gut barrier damage: Apoptosis (↑ caspase-3, graph only, no exact fold reported), ROS significantly ↑, graphical data.	*In vivo*; Fluorescence imaging
PS-MPs (67)	Oral	1.22 μm, 60 mg/day, 5 weeks	Intestinal injury: intestinal barrier dysfunction (↓ ZO-1、Occuldin and Claudin1), angiogenesis significantly decreased (↓TGF-β1 and VEGF-A) graph only, no exact fold reported; Oxidative stress (↓ T-AOC、GSH-Px and GSH), inflammation (↑ TNFα、IL-1β and IL-8) and ER (↑ ATF4、ATF6、CHOP and XBP-1), mitochondria Damage (↓ COXIV, β-F1-ATpase and NRF1), graph only, no exact fold reported.	*In vivo*; Not applicable
PS-MPs (70)	Oral	10-150 μm, 200 μg/g, 5 weeks	Intestinal dysbacteriosis (the abundance of Staphylococcus, Parabacteroides, Bacterodides, Muribaculum, and Akkermansia significantly ↓ and the abundance of Lactobacillus, Dubosiella, Blautia and Besulfovibrio significantly ↑, graph only, no exact fold reported); Inflammation (IL-1α、IL-6、IL-9 ↑, and G-CSF、IL-2、IL-5、IP-10 and RANTES ↓), graph only, no exact fold reported.	*In vivo*; Not applicable
PS particles (71)	OG	1-10 μm, ≤34 mg/kg, 4 weeks	No acute health risk	*In vivo* and *vitro*; Fluorescence microscopy

PS-MPs, polystyrene microplastics; PS-NPs, polystyrene nanoplastics; OD, oral (drinking); OG, oral gavage; ROS, reactive oxygen species; ER, endoplasmic reticulum stress; PK, pyruvate kinase; PPARa, peroxisome proliferator-activated receptor alpha; FAS, fatty acid synthase; ChREBP, carbohydrate response element-binding protein; Fat1, fatty acid translocase 1; Fatp2, fatty acid transport protein 2; CS, citrate synthase; PPARg, peroxisome proliferator-activated receptor gamma; ACC, acetyl-coa carboxylase 1; ACL, ATP citrate lyase; Dgat1, diacylglycerol acyltransferase 1; Dgat2, diacylglycerol acyltransferase 2; Gpat, glycerol-3-phosphate acyltransferase; Muc1, mucin 1; Muc2, mucin 2; Klf4, kruppel-like factor 4; Retnlb, resistin like beta; ARG, arginine; TYR, tyrosine; C4, c4 acylcarnitine; C5, c5 acylcarnitine; C6DC, c6 dicarboxylic acylcarnitine; C16, c16 acylcarnitine; C18, c18 acylcarnitine; LEU, leucine; ILE, isoleucine; PRO-OH, hydroxyproline; ORN, ornithine; PHE, phenylalanine; ZO-1, zonula occludens-1; TGF- b1, transforming growth factor-beta 1; VEGF-A, vascular endothelial growth factor-a; T-AOC, total antioxidant capacity; GSH-Px, glutathione peroxidase; GSH, glutathione; TNF a, tumor necrosis factor-alpha; IL-1b, interleukin-1 beta; IL-8, interleukin-8; ATF4, activating transcription factor; ATF6, activating transcription factor 6; CHOP, C/EBP homologous protein; XBP-1, x-box binding protein 1; COXIV, cytochrome c oxidase subunit iv; b-F1-ATPase, beta subunit of f1-atp synthase; NRF1, nuclear respiratory factor 1; IL-1a, interleukin-1 alpha; IL-6, interleukin-6; IL-9, interleukin-9; G-CSF, granulocyte colony-stimulating factor; IL-2, interleukin-2; IL-5, interleukin-5; IP-10, interferon gamma-induced protein 10; RANTES, regulated on activation; normal t cell expressed and secreted; ↑Increase or enhance; ↓Reduce or inhibit.

### 3.6 Cardiotoxicity

Clinical studies have detected nine MPs polymer types within pericardial, myocardial, and left atrial tissues of cardiac surgery patients, indicating MPs can breach multiple physiological barriers to invade enclosed organs (72). Notably, plastic medical instruments used during procedures may introduce exogenous MPs via the circulatory system, suggesting iatrogenic exposure as a distinct contamination source (72). Animal studies reveal that acute maternal inhalation of MPs (20 nm, 1% concentration, 300 μL) leads to cardiac deposition in both dams and offspring (73), whereas oral MP intake (50 mg/mL, 60 nm) showed no significant cardiac accumulation (74). This evidence implies: (i) Inhalation poses higher translocation risks than oral exposure, (ii) Cardiac accumulation exhibits dose-dependent thresholds, and (iii) Particle size governs tissue infiltration selectivity.

Troponin-I and creatine kinase-MB (CK-MB) serve as critical biomarkers of myocardial injury. Studies demonstrate that high-dose MPs (50 mg/L) significantly elevate serum CK-MB and troponin-I levels in rats, inducing cardiomyocyte apoptosis and abnormal collagen deposition (75), confirming MPs-induced cardiac damage. Similarly, when exposed to MPs, 3D cardiac organoids (COs) exhibit elevated oxidative stress, inflammatory responses, apoptosis, collagen accumulation, and myocardial hypertrophy (evidenced by upregulated myosin heavy chain 7B (MYH7B), atrial natriuretic peptide (ANP), brain natriuretic peptide (BNP), and collagen type I alpha 1 chain (COL1A1) expression) (76). Mechanistically, MPs trigger pyroptosis in Wistar rat cardiomyocytes via the NLRP3/Caspase-1 signaling pathway and oxidative stress, thereby exacerbating cardiac injury (77). Furthermore, MPs provoke cardiac fibrosis through Wnt/β-catenin pathway activation and apoptosis driven by oxidative stress, culminating in cardiovascular toxicity (75). Neonatal rat cardiomyocytes exposed to MPs display reduced mitochondrial membrane potential (ΔΨm) and impaired glycolytic homeostasis, ultimately causing contractile dysfunction and electrophysiological abnormalities (78). Thus, MPs induce cardiac functional decline and electrophysiological disturbances by promoting oxidative stress, mitochondrial dysfunction, and fibrosis (Table 6).

**Table 6:** Comprehensive summary of microplastic induced cardiotoxicity.

Type of MP	Exposure route	Size/Dose/Times	Detected effect	Study model and analytical techniques
Polystyrene nanobeads (73, 74)	INH	20 nm, 1%, 300 μL	MPs deposition in both cardiac tissue and offspring hearts, graphical data;	*In vivo*; Hyperspectral Darkfield microscopy
PS-MPs (76)	OD	0.5 μm, 50 mg/L, 90 days	Myocardial injury: ↑ collagen deposition, graphical data; Cardiac fibrosis markers (↑TGF-β, α-SMA, Collagen1/3 and fibronectin), graph only, no exact fold reported; Oxidative stress markers: MDA levels increased significantly by 1.7%, SOD, GSH-PX, and CAT decreased by 0.49, 53.4 and 8.3% respectively; Apoptosis markers: Bax levels increased by 36%, Bcl-2 levels decreased by 22%; Wnt/β-catenin pathway activated, graph only, no exact fold reported.	*In vivo*; Transmission electron microscopy
PS-MPs (77)	IT	1 μm, 25 and 50 μg, 4 weeks	Myocardial fibrosis, graph only, no exact fold reported; Oxidative stress, inflammation and apoptosis markers: (SOD, TNF-α and caspase-3 ↑).	*In vivo*; Transmission electron microscopy
PS-MPs (71)	OD	0.5 mm, 5 and 50 mg/L, 90 days	Myocardial injury: (↑ CK-MB and cTnl), graph only, no exact fold reported; Cardiac hypertrophy (↑ MYH7B, ANP, BNP, COL1, graph only, no exact fold reported); Oxidative stress markers: (↓SOD, GSH-Px, CAT and ↑ MDA), pyroptosis: (↑ ASC, cleaved GSDMD、IL-1β and IL-18), NLRP3/Caspase-1 pathway activated, graph only, no exact fold reported.	*In vitro* and *vivo*; Transmission electron microscopy

PS-MPs, polystyrene microplastics; OD, oral (drinking); INH, inhalation; IT, intratracheal instillation; ROS, reactive oxygen species; TGF-β, transforming growth factor-β; α-SMA, α-smooth muscle actin; TNF-α, tumor necrosis factor-α; SOD, superoxide dismutase; NLRP3, NOD-like receptor thermal protein domain associated protein 3, MDA, malondialdehyde, GSH-PX, glutathione peroxidase, CAT, catalase, Bax, Bcl-2 associated x protein, Bcl-2, b-cell lymphoma 2, caspase-3, caspase-3, CK-MB, creatine kinase mb isoenzyme, cTnl, cardiac troponin I, MYH7B, myosin heavy chain 7b, ANP, atrial natriuretic peptide, BNP, brain natriuretic peptide, COL1, collagen type 1, ASC, apoptosis-associated speck-like protein containing a caspase recruitment domain, GSDMD, gasdermin D, IL-1β, interleukin-1 beta, IL-18, interleukin-18, caspase-1, caspase-1, ↑increase or enhance; ↓reduce or inhibit.

### 3.7 Reproductive toxicity

The reproductive toxicity of MPs and NPs represents a critical nexus linking systemic multi-organ toxicity with population-level and long-term health risks. Given the extensive evidence that MPs/NPs affect multiple organs—including the gut, liver, immune system, and nervous system—the reproductive system is uniquely important because it serves as both a direct target of toxicity and a conduit for intergenerational transmission. This section therefore integrates evidence across scales, beginning with direct toxic effects on male and female reproductive organs, and extending to developmental and intergenerational outcomes in offspring. In particular, we contextualize nervous system and metabolic impairments in progeny as part of a broader multi-organ developmental toxicity framework, rather than isolated neurodevelopmental endpoints. By doing so, this section aims to clarify how parental exposure—predominantly via oral routes—can translate into systemic and heritable effects mediated by particle translocation, oxidative stress, endocrine disruption, and epigenetic reprogramming.

This section systematically reviews the direct adverse effects of MPs/NPs on the reproductive system and their transgenerational consequences. We first detail toxicity and underlying mechanisms in male and female reproductive systems, focusing on gametogenesis, steroidogenesis, and gonadal integrity. Given the prevalence of maternal exposure, its potential intergenerational health effects are of particular significance. Therefore, we further discuss how parental exposure influences offspring development across multiple organ systems, including the nervous, metabolic, hepatic, and reproductive systems, thereby extending the scope of MPs toxicity from an individual lifespan to a transgenerational scale.

#### 3.7.1 The impact of microplastics on the male reproductive system

The testis, composed of seminiferous tubules and interstitial tissue, primarily facilitates spermatogenesis and testosterone secretion (79). Studies reveal that exposure to MPs (1 μm, 1 mg/kg, 5 mg/kg) and NPs (100 nm, 1 mg/L, 10 mg/L) reduces germinal epithelium stratification, disrupts spermatogenic cell alignment, and exacerbates interstitial fibrosis (80, 81). In contrast, epididymal tissue shows no significant apoptosis, epithelial disorganization, or barrier dysfunction following MPs exposure (82), indicating greater testicular sensitivity to MPs. The blood-testis barrier (BTB)—formed by Sertoli cell tight junctions, basement membrane, and tunica propria—is critical for maintaining the spermatogenic microenvironment and immune privilege (83). Research has found that MPs (250–1,000 mg/L) induce Sertoli cell death in porcine testes, consequently compromising BTB integrity (84). Ultrastructural analyses demonstrate that both MPs (0.5–5 μm) and NPs (80 nm) cause mitochondrial swelling, disrupted intercellular junctions, and cytoplasmic edema in Sertoli cells, with nanoscale particles inducing more severe damage (85). Regarding spermatogenesis, NPs (1 mg/L) interfere with meiotic progression in spermatocytes by inducing mitochondrial vacuolization and cristae disintegration, while medium-to-high concentrations (1.0–10.0 mg/kg) significantly increase incidence of acrosomal malformations (e.g., asymmetrical or punctate structures) in mature sperm (85, 86). Collectively, MPs impair male fertility by disrupting BTB integrity, directly damaging mitochondrial function in germ cells, and compromising acrosome development.

Mechanistic studies on MPs-induced male reproductive toxicity reveal that MPs disrupt sperm energy metabolism by triggering oxidative stress to activate the c-Jun N-terminal kinase/p38 mitogen-activated protein kinase (JNK/p38 MAPK) pathway. This damage and impaired testosterone secretion were counteracted by the ROS scavenger N-acetylcysteine (NAC) or the p38 MAPK inhibitor SB203580 (87). Concurrently, MPs inhibit the nuclear factor erythroid 2-related factor 2/heme oxygenase-1(Nrf2/HO-1) antioxidant system, promoting NF-κB nuclear translocation and pro-inflammatory cytokine release (IL-1β, IL-6), thereby exacerbating the testicular inflammatory microenvironment and reducing viable sperm counts (88). Further research demonstrates that MPs activate hypoxia-inducible factor 1-alpha (HIF-1α) expression in mouse TM3 Leydig cells and testicular tissue via the extracellular signal-regulated kinase 1/2 (ERK1/2)/MAPK and AKT pathways, down-regulating steroidogenic acute regulatory protein (StAR) expression. This directly suppresses testosterone synthesis and damage’s reproductive function (89). Thus, MPs impair spermatogenesis and diminish male reproductive capacity by disrupting redox homeostasis and aggravating testicular inflammation (Table 7).

**Table 7:** Comprehensive summary of microplastic induced reproductive toxicity.

Type of MP	Exposure route	Size/Dose/Times	Detected effect	Study model and analytical techniques
PS-MPs (80)	OD	1 μm, 1 mg/kg and 5 mg/kg, 4 weeks	Ca^2+^/ROS/NF-κB signaling axis activated; Premature testicular senescence (α-SMA, p21, p16 and p53 significantly ↑, graph only, no exact fold reported).	*In vitro* and *vivo*; Not applicable
PS-NPs (81)	OD	100 nm, 1 mg/L and 10 mg/L, from GD 0 to PND 21	Epididymal sperm count significantly ↓ (27.08 and 55.28%); Absolute testicular weight significantly ↓ 23.38 and 55.28% on PND 21 and 18.58% &15.86% on PND 56; Testicular oxidative stress in the offspring (MDA↑, CAT and SOD↓, graph only, no exact fold reported).	*In vivo*; Not applicable
PS-MPs (87)	OG	5.0–5.9 μm, 1 mg/day, 42 day	Testicular oxidative stress (ROS and MDA significantly ↑, GSH significantly ↓, graph only, no exact fold reported); JNK/p38 MAPK pathway activation; Sperm energy metabolism damage (SDH and LDH significantly ↓, graph only, no exact fold reported).	*In vivo*; Not applicable
PS-MPs (85)	OD	0.5 and 5 μm, 1 mg/L, 12 weeks	Sertoli cell numbers significantly ↓, graph only, no exact fold reported; Leydig cell area significantly ↓, graph only, no exact fold reported.	*In vitro* and *vivo*; Not applicable
PS-NPs (86)	Oral	50 nm, 1.0 and 10.0 mg/kg, 35 days; 50 μg/mL, 100 μg/mL, 200 μg/mL for 24 h	Autophagy (LC3B-II and Beclin1 significantly ↓, graph only, no exact fold reported); Sperm acrosomal malformation significantly ↑, graph only, no exact fold reported.	*In vitro* and *vivo*; Biofluorescence imaging assay
PS-MPs (88)	OD	5 μm, 0.01, 0.1 and 1 mg/day, 35 days	Nrf2/HO-1/NF-κB pathway activated; Nrf2 significantly ↓; Testicular inflammatory (IL-1β and IL-6 significantly↑, graph only, no exact fold reported); Abnormal sperm quality, graph only, no exact fold reported.	*In vivo*; Not applicable
PS-NPs (89)	IV	20 nm, 50 μg/kg/day, 2 days	ERK1/2 /MAPK and AKT pathways activation; p-ERK and p-AKT significantly ↑, Testosterone ↓ and Reproductive function significantly ↓, graph only, no exact fold reported.	*In vivo*; Not applicable
PS-MPs (91, 94)	Oral	5 μm, 0.1 mg/day, four estrus cycles	Ovarian oxidative stress (MDA and CAT ↑, PSH ↓, graph only, no exact fold reported); Ovarian dysfunction.	*In vivo*; Fluorescence microscopy
PS-MPs (92)	Oral	5-10 μm, 100 mg/L, 35 days, 500 mg/L for 24 h	ROS (HO-1 and iNOS↑; GPX1 and SOD1↓); The number of follicles significantly ↓, graph only, no exact fold reported; Granulosa cell cycle arrest and necrosis (p53, p21, RIPK1, RIPK3 and MLKL↑, Cyclin B/D/E ↓, graph only, no exact fold reported).	*In vitro* and *vivo*; Not applicable
PS-NPs (96)	OG	20 nm, 1 mg/day, 5 weeks; 100 μg/mL for 48 h	Apoptosis (BAX↑ and Bcl2 significantly ↓, graph only, no exact fold reported); Fertility significantly ↓, graph only, no exact fold reported.	*In vitro* and *vivo*; Fluorescence microscopy
PS-MPs (97)	OG	1 μm, 2 mg/kg, 28 days	PERK-eIF2α-ATF4-CHOP pathway activated; ROS (SOD and CAT ↓, MDA ↑, graph only, no exact fold reported); Ovarian apoptosis (Bax↑ and Bcl-2 ↓ and graph only, no exact fold reported).	*In vivo*; Not applicable
PS-MPs (98)	OD	0.5 μm, 1.5 mg/kg/day, 90 days	ROS (SOD and CAT ↓, MDA ↑, graph only, no exact fold reported); NLRP3/Caspase-1 pathway activation; Granulosa pyroptosis and apoptosis (Cleaved-Caspase-3 significantly ↑, graph only, no exact fold reported).	*In vitro* and *vivo*; Transmission electron telescope
PS-MP (93)	OD	5-10 μm, 100 mg/L, 42 days; 1, 5, 25 μg/mL for 20 min	TLR4/NOX2 signaling axis activated; ROS (8-OH DG↑, SOD and GPx1 significantly ↓, graph only, no exact fold reported); Ovarian fibrosis (COL1 and α-SMA significantly ↑, graph only, no exact fold reported).	*In vitro* and *vivo*; Not applicable
PS-MPs (94)	OD	0.5 μm, 0.15 and 1.5 mg/day, 90 days; 0、1、5、25 μg/mL for 20 min	ROS (SOD, GSH-PX and CAT significantly ↑, MDA significantly ↓, graph only, no exact fold reported); Granulosa apoptosis (Bax ↑ and Bcl-2 ↓, graph only, no exact fold reported); Wnt/β-catenin pathway activated; Ovarian fibrosis ↑;	*In vitro* and *vivo*; Transmission electron microscopy
PS-MPs and PS-NPs (99)	OD	1,000 nm, 1 mg/day, 17 days	The TAC level significantly ↓; The proportion of apoptotic cells significantly ↑; Offspring anxiety-like behavior significantly↑; graph only, no exact fold reported.	*In vivo*; Multispectral FX PRO system
PS-NPs (7)	Oral	50 nm, 0.5-1000 μg/day, GD8 to PND14	Neural stem cell dysfunction: (Ki67^+^ proliferative cells significantly ↓, graph only, no exact fold reported); The thickness of the neuronal layer in the CA3 region significantly ↓, graph only, no exact fold reported.	*In vitro* and *vivo*; Fluorescence microscopy
PS-MPs (102)	OD	0.5 or 5 μm, 100 or 1,000 μg/L, GD to PND	Metabolism dysfunction (TC and LDL-C significantly ↑, TG and HDL-C ↓ in male; TG significantly ↓ in female, graph only, no exact fold reported).	*In vivo*; Not applicable
PS-MPs (102)	OD	5 μm, 100 and 1,000 μg/L, 6 weeks	Metabolism dysfunction (TC and TG significantly ↑, graph only, no exact fold reported); The OCTN2 transporter expression significantly ↓, graph only, no exact fold reported; CPT1/CPT2 enzyme activity imbalance, graph only, no exact fold reported.	*In vivo*; Not applicable
DCHP (104)	OG	10 mg/kg, 4 weeks	Metabolism dysfunction (GTT and ITT significantly ↑, graph only, no exact fold reported).	*In vivo*; Not applicable
PS-MPs (105)	OD	0.5 μm, 0.5, 5 and 50 mg/L, GD to PND	Offspring testis weight significantly ↓, graph only, no exact fold reported; Sex hormone (Testosterone and INH-B significantly ↓, graph only, no exact fold reported); Sperm count and motility significantly ↓, graph only, no exact fold reported; Reproductive function significantly ↓, graph only, no exact fold reported.	*In vivo*; Not applicable

PS-MPs, polystyrene microplastics; PS-NPs, polystyrene nanoplastics; OD, oral (drinking); OG, oral gavage; IV, tail vein injection; ROS, reactive oxygen species; GTT, glucose tolerance test; ITT, insulin tolerance test; CPT1, carnitine acyl transferase 1; CPT2, carnitine acyl transferase 2; OCTN2, organic cation/carnitine transporter 2; TC, total cholesterol; TG, triglycerides; LDL-C, low-density lipoprotein cholesterol; HDL-C, high-density lipoprotein cholesterol; NF-kB, nuclear factor kappa-B; α-SMA, α-smooth muscle actin; PND, postnatal day; JNK, c-Jun N-terminal kinase; p38 MAPK, p38 mitogen-activated protein kinase; Nrf2, Nuclear factor erythroid 2-related factor 2; HO-1, Heme oxygenase 1; IL-1β, interleukin-1β; IL-6, interleukin-6; SOD, superoxide dismutase; PSH, protein sulfhydryl; MDA, malondialdehyde; CAT, catalase; TLR4, toll-like receptor 4; NOX2, NADPH oxidase 2; Akt, protein kinase B; NLRP3, NOD-like receptor thermal protein domain associated protein 3; Bax, BCL-2-associated X protein; Bcl-2, B-cell lymphoma-2; ERK, extracellular regulated protein kinases; MAPK, mitogen-activated protein kinases, ↑Increase or enhance; ↓Reduce or inhibit.

#### 3.7.2 The impact of microplastics on the female reproductive system

Studies confirm that the reproductive toxicity of MPs in female mammals is dose-dependent, with significantly more pronounced damaging effects on the ovaries and uterus than on the male reproductive system (90). Animal experiments reveal that female rats orally exposed to 5-μm MPs exhibit particle accumulation in ovarian tissue, accompanied by reduced ovarian weight, decreased serum estradiol levels, and disrupted estrous cycles (91). Pathological analyses further demonstrate that MPs exposure induces cell cycle arrest and necrosis in ovarian granulosa cells (92), while concurrently triggering endometrial thinning and abnormal collagen fiber deposition. These findings suggest MPs may contribute to diminished ovarian reserve and uterine fibrosis (93–95).

Research demonstrates that MPs exposure suppresses the activity of ovarian antioxidant enzymes (SOD, CAT, GSH-Px), leading to the accumulation of ROS and malondialdehyde (MDA) (91, 96). Wu et al. further revealed that MPs promote excessive ROS generation by triggering the cannabinoid receptor 1/cereblon/Yin Yang 1/cytochrome P450 2E1(CNR1/CRBN/YY1/CYP2E1) signaling axis in ovarian granulosa cells, consequently inducing oxidative DNA damage (92). Moreover, MPs-induced ovarian toxicity in juvenile rats correlates with oxidative stress and activation of the protein kinase R-like endoplasmic reticulum kinase-eukaryotic initiation factor 2α-activating transcription factor 4-C/EBP homologous protein (PERK-eIF2α-ATF4-CHOP) signaling pathway (97). Notably, oxidative stress inhibitors (AM251 or DAS) reversed PS-triggered ovarian damage (92, 97). Excess ROS subsequently upregulates NLRP3/Caspase-1 pathway components and Cleaved-Caspase-3 expression, provoking pyroptosis and apoptosis in granulosa cells, which underlies MPs reproductive toxicity (98). These findings collectively establish oxidative stress as the central mechanism in MPs-induced ovarian injury and reproductive impairment.

Uterine fibrosis represents a major factor in female reproductive dysfunction. Studies indicate that MPs mediate oxidative stress via the TLR4/ NADPH oxidase 2 (NOX2) signaling axis, subsequently activating Notch and TGF-β pathways to drive ovarian fibrosis and uterine collagen deposition (93). MPs exposure also initiates fibrosis by markedly upregulating Wnt/β-catenin signaling, TGF-β, and α-smooth muscle actin (α-SMA) expression, triggering granulosa cell apoptosis (94). Additionally, MPs disrupt cytoskeletal protein expression (α-tubulin, Dishevelled-associated activator of morphogenesis 1(DAAM-1)) and interfere with hippo pathway activity, inhibiting granulosa cell proliferation and steroidogenesis, ultimately diminishing ovarian reserve and fertility (91, 96). Thus, oxidative stress, inflammatory signaling, and fibrotic pathways constitute key molecular networks through which MPs compromise female reproductive function (Table 7).

#### 3.7.3 Intergenerational toxicity of microplastics

Parental exposure to MPs and NPs induces transgenerational toxicity that extends beyond direct reproductive impairment and reflects systemic developmental vulnerability across multiple organs. While early studies emphasized neurodevelopmental outcomes, emerging evidence indicates that offspring toxicity involves coordinated disturbances in the nervous, metabolic, hepatic, endocrine, and reproductive systems, driven by shared upstream mechanisms such as oxidative stress, inflammation, endocrine disruption, and epigenetic modification.

Gestational maternal MPs exposure has been shown to induce anxiety-like behaviors in offspring by suppressing *γ*-aminobutyric acid (GABA) synthesis in the prefrontal cortex and amygdala. This neural impairment is reversible by N-acetylcysteine (NAC) antioxidant intervention, indicating oxidative damage as the core driver of offspring neurotoxicity and anxiety phenotypes (99). Concurrently, maternal MPs exposure during embryonic and early postnatal stages disrupts neural stem cell function, leading to aberrant brain development in offspring (7). These findings position neurodevelopmental impairment as a sensitive, but not exclusive, endpoint of intergenerational MPs toxicity.

Crucially, nervous system effects occur alongside dysfunction in other organ systems. MPs/NPs can translocate across biological barriers, including the placental barrier and mammary epithelium, enabling direct fetal and neonatal exposure. In mammals, NPs have been detected in placental tissue, fetal organs, and breast milk, while in aquatic organisms, particle transfer occurs via gametes, facilitating multigenerational bioaccumulation (100). In aquatic models like zebrafish and Daphnia, NPs exposure results in particle transfer to unexposed generations, causing oxidative stress, inflammation, and DNA damage through mechanisms such as ROS-induced ROS release (RIRR) and mitochondrial dysfunction (101). Key signaling pathways involved include the Nrf2 pathway, which modulates antioxidant responses; the Wnt/β-catenin pathway, affecting development; and the NF-κB pathway, promoting inflammation and apoptosis (100). Epigenetic modifications, such as DNA hypomethylation and histone alterations, have been observed in nematodes and fish, suggesting heritable changes that exacerbate toxicity across generations (101).

Beyond neurodevelopment, maternal MPs exposure disrupts offspring metabolic homeostasis. Exposure during gestation and lactation reduces prenatal and postnatal body weights in offspring. High-dose MPs (10 mg/L) additionally decrease liver weights in male progeny, concomitant with hepatic oxidative stress, inflammatory cell infiltration, upregulated proinflammatory cytokines, glucose metabolism dysregulation, and lipid deposition (102, 103). Paternal MPs exposure similarly alters insulin signaling pathways and metabolic gene expression in offspring livers (104). These metabolic disturbances frequently co-occur with endocrine and hepatic alterations, reinforcing the concept of multi-organ developmental toxicity.

Intergenerational reproductive effects have also been documented. Maternal MPs exposure during critical developmental windows reduces testicular weight and sperm count in male offspring and impairs reproductive capacity in subsequent life stages (103, 105). In aquatic models, co-exposure with chemicals like heavy metals or organic pollutants synergistically enhances thyroid disruption and growth impairments (101). Epigenetic alterations, including DNA hypomethylation and histone modification, have been identified in nematodes and fish, suggesting heritable molecular reprogramming that amplifies toxicity across generations (101).

Collectively, available evidence demonstrates that intergenerational toxicity of MPs/NPs reflects integrated multi-organ developmental disruption rather than isolated nervous system effects. These findings underscore the necessity of incorporating organ crosstalk, exposure timing, particle characteristics, and epigenetic inheritance into future risk assessment frameworks (Table 7).

### 3.8 Common cellular and molecular mechanisms of microplastics-induced toxicity

Although microplastics-induced toxicity manifests in an organ-specific manner, converging evidence indicates that several shared cellular and molecular mechanisms underlie these diverse pathological outcomes. Across multiple organ systems, oxidative stress emerges as a central initiating event, characterized by excessive ROS generation, mitochondrial dysfunction, and impaired antioxidant defenses. This oxidative imbalance subsequently activates inflammatory signaling pathways, particularly NF-κB and NLRP3 inflammasome signaling, resulting in sustained cytokine release and chronic tissue inflammation.

**Figure 1:**
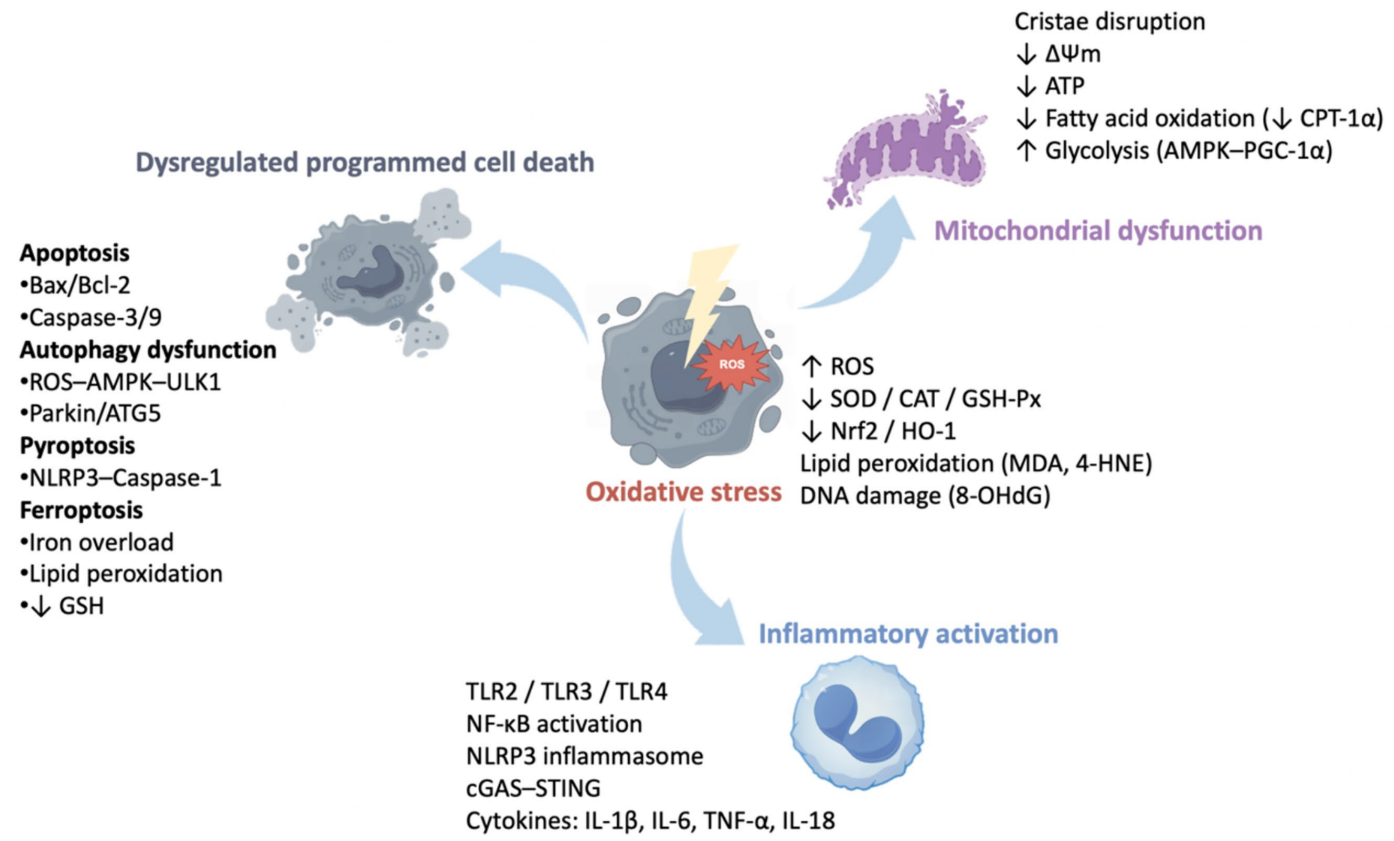
Cellular and molecular mechanisms underlying microplastics-induced toxicity and organ crosstalk.

In parallel, microplastics disrupt intracellular homeostasis by dysregulating programmed cell death pathways, including apoptosis, autophagy, pyroptosis, and ferroptosis, depending on particle size, exposure route, and tissue context. Emerging evidence further highlights endocrine disruption, metabolic reprogramming, and epigenetic alterations as critical contributors to both systemic and transgenerational toxicity. Importantly, these mechanisms do not operate in isolation but are amplified through inter-organ communication axes such as the gut–liver–brain axis, ultimately driving systemic toxicity. As illustrated in the Figure 1, oxidative stress, mitochondrial dysfunction, inflammatory activation, and dysregulated cell death represent convergent mechanisms across multiple organs.

### 3.9 Systemic effects and cross-organ crosstalk

The preceding sections have described the organ-specific injuries induced by MPs in the gut, liver, brain, lung, and other tissues. However, these toxic effects do not occur in isolation. Instead, accumulating evidence indicates that MP-induced toxicity frequently manifests through coordinated multi-organ communication, in which localized tissue injury is amplified and transmitted via defined physiological axes. Recognizing these cross-organ interactions is therefore essential for understanding how MP exposure leads to systemic pathologies that extend well beyond single-organ dysfunction.

Among these axes, the gut–liver axis represents a primary conduit through which intestinal injury propagates systemic toxicity. As discussed earlier, MPs disrupt intestinal barrier integrity and induce microbial dysbiosis. These local intestinal alterations are accompanied by activation of the Wnt/β-catenin pathway, resulting in increased epithelial permeability and enhanced translocation of lipopolysaccharide (LPS) and microbial metabolites (e.g., secondary bile acids) into the portal circulation. The subsequent delivery of these signals to the liver triggers hepatic inflammation and oxidative stress (5, 6). In parallel, MPs disturb the bile acid-short-chain fatty acid metabolism axis, leading to suppression of farnesoid X receptor (FXR) signaling and thereby promoting hepatic lipid accumulation and insulin resistance (106, 107). Consistent with these findings, further studies demonstrate that MPs activate hepatic Kupffer cells via the TLR2/NF-κB/NLRP3 signaling pathway, stimulating the release of proinflammatory cytokines, including IL-1β and TNF-α, which further exacerbate liver injury (64). Notably, airborne particulate MPs may also induce hepatotoxicity by disrupting the "airway microbiota-lung-liver” axis (108), suggesting that hepatic injury can arise from both intestinal and pulmonary sources of MP exposure.

Importantly, the gut–liver axis does not operate in isolation but can further extend its influence to the central nervous system. Integrating the neurological and hepatic effects described in previous sections, recent evidence supports the existence of a gut–liver–brain axis through which MPs-induced intestinal and hepatic dysfunction converge to impact brain function. Specifically, circulating microbial and metabolic intermediates, including tryptophan-derived metabolites, have been implicated in mediating MP-triggered neurobehavioral abnormalities (6). Preclinical studies demonstrate that activation of this axis induces anxiety-like behaviors in experimental animals while simultaneously aggravating hepatic metabolic dysregulation (6), highlighting a shared mechanistic basis underlying liver–brain comorbidity following MP exposure.

In parallel, direct gut–brain communication represents another critical pathway linking intestinal injury to neurotoxicity. MPs exposure significantly reduces colonic mucin secretion in mice, leading to compromised gut barrier function and a pronounced reduction in gut microbiota α-diversity, characterized by the abnormal proliferation of opportunistic pathogens such as Mesorhizobium and Lwoffii. Concurrently, MPs upregulate interleukin-17C (IL-17C) expression in intestinal epithelium. This cytokine is capable of penetrating the blood–brain barrier, where it promotes parenchymal inflammation, neuronal damage, and significant impairments in social behavior (109, 110). In addition, NPs induce lysosomal damage in intestinal macrophages, triggering aberrant interleukin-1 (IL-1) secretion that activates microglia and drives T helper 17 (Th17) cell differentiation, ultimately compromising cognitive function and short-term memory (60). MPs have also been shown to activate the macrophage very late antigen 4-vascular cell adhesion molecule 1 (VLA4-VCAM1) signaling axis while concurrently altering intestinal bile acid and carbohydrate metabolic profiles. Together, these effects impair intestinal motility, reduce water absorption, and induce depressive-like phenotypes in experimental models (111). Additional neurotoxic mechanisms associated with gut–brain axis dysfunction include MP-induced disruption of hippocampal circadian rhythms and altered neurotransmitter metabolism (112, 113).

Collectively, the studies summarized above demonstrate that the adverse health effects of MPs are not simply the cumulative outcome of isolated organ injuries. Rather, they emerge from dynamic, networked interactions along defined physiological axes, such as the gut–liver and gut–liver–brain axes. Within this axis-centered framework, MPs initiate localized damage—often in barrier or metabolically active organs such as the gut and lung—which is subsequently propagated to distant organs through circulating immune mediators, microbial metabolites, and neural signaling pathways. This integrative perspective provides a mechanistic bridge between organ-specific toxicity and systemic pathology and underscores the potential risk of environmental MPs exposure to liver and nervous system function in social mammals, including humans (Table 8).

**Table 8:** Comprehensive summary of microplastic induced systemic effects and cross-organ crosstalk.

Type of MP	Exposure route	Size/Dose/Times	Detected effect	Study model and analytical techniques
PGA-MPs (6)	EP	687.5 nm, 1 and 100 mg/L, 28 days	Anxiety-like behaviors significantly ↑; Brain inflammation (IL-1β, TNF-α and IL-10 significantly ↑, graph only, no exact fold reported); Changes in intestinal microbiota significantly ↑, graph only, no exact fold reported; Liver dysfunction (ALT and LPS ↑, graph only, no exact fold reported); Inflammation (TNF-α and IL-10 ↑, graph only, no exact fold reported); Metabolic disorder (TC, TG↑, graph only, no exact fold reported); Apoptosis (caspase3, p53 and Bax↑, Bcl-2↓, graph only, no exact fold reported).	*In vivo*; Not applicable
PS-MPs (106)	OG	10 μm, 10 mg/kg/day, 28 days	Liver ROS (Nrf2, SOD1, SOD2, HO-1 significantly ↓, graph only, no exact fold reported); Liver inflammation (TNF-α and IL-6 significantly ↑, graph only, no exact fold reported); Changes in intestinal barrier function (ZO-1 significantly ↓, graph only, no exact fold reported).	*In vivo*; Not applicable
PS-MPs (107)	OD	1 μm, 10 mg/L, 1 or 2 weeks	ROS (CAT, SOD and GSH-Px significantly ↓, graph only, no exact fold reported); Insulin resistance (FBG, insulin and HOMA-IR significantly ↑, graph only, no exact fold reported).	*In vivo*; Not applicable
PS-MPs and PS-NPs (108)	INH	5 μm, 0.03 mg, 42 day	Liver function and disrupt serum antioxidant (AST and ALT ↑, SOD↓, graph only, no exact fold reported); Changes in intestinal and nasal microbiota; Liver transcriptomic changes, graph only.	*In vivo*; Not applicable
PS-NPs (109)	OG	50 nm, 20 mL/kg, 28 days	Mycoplasma and Coriobacteriaceae proliferation significantly ↑, graph only, no exact fold reported; Intestines IL-17C significantly ↑, graph only, no exact fold reported; Inflammation and significantly ↑; Brain damage.	*In vivo*; Not applicable
PS-MPs (110)	OD	50 μm, 100 μg/L, 10 weeks	Social competence significantly ↓, graph only, no exact fold reported; Changes in intestinal microbiota (abundance of Actinobacteria and Bifidobacterium ↓, Tyzzerella ↑); Brain Oxytocin significantly ↓, graph only, no exact fold reported.	*In vivo*; Not applicable
PS-MPs (111)	OD	0.1、5 and 50 μm, 10 mg/L, 120 days	Colitis significantly ↑, graph only, no exact fold reported; Balance of the gut microbiome significantly disrupted; Number of microglia significantly ↓, graph only, no exact fold reported; Change in bile acid and carbohydrate metabolism;	*In vivo*; Not applicable
PS-NPs (112)	OD	80 nm, 60 μg/day, 42 days	Neuronal damage (BDNF and CREB ↓, graph only, no exact fold reported); Colon injury (Occludin, Claudin-1 and ZO-1 significantly ↓, graph only, no exact fold reported); Gut microbiota dysbiosis and metabolic disorders.	*In vivo*; Fluorescence imaging
PS-MPs (113)	OG	1 μm, 0.5 mg/day, 60 days	Anxiety-like behaviors significantly ↑, graph only, no exact fold reported; Intestinal permeability significantly ↑, graph only, no exact fold reported; Gut microbiota dysbiosis.	*In vivo*; Not applicable

PS-MPs, polystyrene microplastics; PS-NPs, polystyrene nanoplastics; OD, oral (drinking); OG, oral gavage; INH, inhalation; EP, environmental exposure; ROS, reactive oxygen species; IL-17C, interleukin 17C; ALT, alanine transaminase; LPS, lipopolysaccharide; TC, total cholesterol; TG, triglycerides; Bax, Bcl-2 associated x protein; Bcl-2, b-cell lymphoma 2; Nrf2, nuclear factor erythroid 2-related factor 2; SOD1, superoxide dismutase 1; SOD2, superoxide dismutase 2; Ho-1, heme oxygenase-1; IL-6, interleukin-6; ZO-1, zonula occludens-1; CAT, catalase; GSH-Px, glutathione peroxidase; HOMA-IR, homeostasis model assessment of insulin resistance; AST, aspartate transaminase; SOD, superoxide dismutase; BDNF, brain-derived neurotrophic factor; CREB, camp response element-binding protein; Claudin-1, claudin-1; IL-1b, interleukin-1 beta; TNF-a, tumor necrosis factor-alpha; IL-10, interleukin-10; ↑Increase or enhance; ↓Reduce or inhibit.

A growing body of preclinical evidence has demonstrated that microplastics can accumulate in multiple organs—including the liver, nervous system, lungs, kidneys, intestines, heart, and reproductive system—and induce multi-organ toxicity, indicating their broad organ targeting potential. Commonly used exposure routes primarily include the respiratory system (e.g., inhalation, intratracheal instillation, and intranasal administration) and the digestive system (e.g., drinking water and oral ingestion). Respiratory exposure tends to cause localized lung injury, whereas digestive exposure may facilitate the transport of microplastics to distant organs via systemic circulation, leading to toxic effects in the liver, nervous system, kidneys, intestines, heart, and reproductive system. However, current research still exhibits several important limitations. First, most studies lack specific and quantitative methods for detecting microplastics within organs; conventional techniques likely underestimate their actual accumulation levels. Therefore, there is an urgent need to develop ultrasensitive, cost-effective, and efficient detection technologies suitable for complex biological matrices to advance the field. Second, substantial variations in exposure doses, durations, and particle sizes across studies have led to low comparability and even inconsistent conclusions. For instance, one study showed that high-dose (200 μg/g), larger-sized (10–150 μm) microplastic exposure for five weeks significantly upregulates the expression of TLR4, activator protein 1(AP-1), and interferon regulatory factor 5 (IRF5), and induces immune cell infiltration in the colon and duodenum (70). In contrast, another study found that short-term (4 weeks) exposure to low-dose (≤34 mg/kg), smaller-sized (1–10 μm) microplastics did not elicit significant inflammatory responses in the same intestinal segments (71). Such heterogeneity underscores the urgency of standardizing experimental parameters. Moreover, many toxicological studies employ high doses of microplastics to accelerate the manifestation of toxicity, but these exposure levels often far exceed realistic environmental concentrations, thereby limiting the physiological and ecological relevance of the findings. Future research should focus more on low-dose exposures that reflect actual environmental pollution levels and further explore the combined effects of microplastics with varying properties to more accurately assess their risks to human health and ecosystems.

## 4 Conclusion and prospect

Microplastics can enter the organism through the respiratory system (e.g., inhalation, intratracheal instillation, and intranasal administration) and the digestive system (e.g., drinking water and oral ingestion), subsequently accumulating in multiple organs and inducing toxic effects in the liver, nervous system, lungs, kidneys, intestines, heart, and reproductive system. This review systematically summarizes the underlying mechanisms of microplastic-induced organ toxicity, highlighting that oxidative stress, inflammatory response, mitochondrial dysfunction, and autophagy are common pathological processes across various organ injuries. Beyond these direct toxicological effects, emerging evidence suggests that parental exposure to microplastics may result in significant transgenerational effects. While the genetic and epigenetic mechanisms (e.g., DNA methylation, histone modifications) mediating these effects remain largely unknown, they are hypothesized to be crucial in perpetuating toxicity across generations. Furthermore, microplastics may mediate systemic toxicity through inter-organ communication networks such as the gut-liver axis, lung-liver axis, and gut-liver-brain axis. By synthesizing organ-specific injury with cross-organ communication networks and developmental toxicity, this review moves beyond descriptive toxicology and proposes a mechanistic paradigm for understanding microplastics-induced systemic toxicity. The graphical abstract provides an integrated visual summary linking exposure routes, shared molecular mechanisms, organ crosstalk, and transgenerational effects.

Future research should focus on elucidating how microplastics interfere with inter-organ signaling, metabolite transport, and immune communication, leading to multi-organ toxicity. *In vitro* co-culture models and integrated multi-organ platforms *in vivo* could be employed to further unravel these complex interactions. A deeper understanding of the molecular and physiological pathways through which microplastics induce organ damage is crucial for developing strategies to mitigate their risks to human health. It is noteworthy that inflammation and oxidative stress represent core mechanisms in microplastic-induced multi-organ injury. Exercise, as an effective intervention with antioxidant and anti-fibrotic properties, could be explored in future studies as a lifestyle-based approach to attenuate microplastic-related toxic effects. Additionally, certain nutritional supplements (such as anthocyanins and resveratrol), known for their antioxidant and anti-inflammatory properties, may serve as potential adjuvant strategies for alleviating multi-organ damage caused by microplastics and warrant further investigation.
